# SGLT2 inhibitor prescription at discharge in very old adults with heart failure irrespective of diabetes status^☆^

**DOI:** 10.1016/j.ijcha.2026.101980

**Published:** 2026-07-30

**Authors:** Rémi Esser, Marc Harboun, Marine Larbaneix, Alejandro Mondragon, Sophie Balesdent, Marlène Esteban, Jennifer Corny, Christine Farges, Vincenzo Palermo, Nicolas Pages, Sophie Nisse Durgeat, Olivier Maurou

**Affiliations:** a Department of Cardiogeriatrics, Hôpital La Porte Verte, Versailles, France; b Department of Pharmacy, Hôpital La Porte Verte, Versailles, France; c Department of Cardiology, Hôpital Marie Lannelongue, Le Plessis Robinson, France; d NP MEDICAL, Medical Affairs, Bordeaux, France

**Keywords:** Heart failure, SGLT2 inhibitors, Older adults, Cardiogeriatrics, Frailty

## Abstract

**Background:**

Sodium–glucose cotransporter 2 inhibitors (SGLT2i) are recommended across the heart failure (HF) spectrum, irrespective of diabetes status. However, prescribing patterns in very old adults with multimorbidity, renal dysfunction, and geriatric vulnerability remain insufficiently characterised.

**Methods:**

We conducted a single-centre retrospective cross-sectional study in a specialised acute cardiogeriatric unit between January 1, 2025, and February 13, 2026. Consecutive hospitalisations with confirmed HF and documented SGLT2i discharge status were included. The primary outcome was absence of SGLT2i at discharge. Multivariable logistic regression identified factors independently associated with non-prescription.

**Results:**

Among 1235 hospitalisations, median age was 88.6 years [IQR 82–92], 54.5% were women, and 32.7% had diabetes. SGLT2i were prescribed in 1016 hospitalisations (82.3%) and absent in 219 (17.7%). Prescription did not differ by diabetes status (80.4% vs 83.2%, *p* = 0.276) or across HbA1c tertiles. In the prespecified primary model, non-prescription was associated with CKD stage 4 (aOR 2.79 [95% CI 1.97–3.96]), CKD stage 5 (aOR 20.29 [9.67–42.56]), palliative orientation (aOR 1.62 [1.09–2.41]), and HFrEF versus HFpEF (aOR 1.55 [1.08–2.22]). Higher albumin and medication count were associated with lower odds of non-prescription. Diabetes, age, and Charlson Comorbidity Index were not independently associated with the primary outcome.

**Conclusion:**

SGLT2i prescription was frequent and similar according to diabetes status and glycaemic control. Advanced CKD and palliative-oriented care were the most consistent correlates of non-prescription. These findings describe prescribing patterns but do not establish the appropriateness of individual decisions.

## 1 Introduction

Heart failure (HF) is a leading cause of hospitalisation, disability, and mortality in older adults [1]. The burden is particularly high in adults aged 75 years and older, particularly among octogenarians and nonagenarians, who account for a substantial proportion of HF admissions in geriatric care settings. This population is characterised by multimorbidity, polypharmacy, frailty, functional decline, cognitive impairment, malnutrition, and chronic kidney disease (CKD), which complicate guideline-directed therapy in routine practice [2], [3].

Sodium–glucose cotransporter 2 inhibitors (SGLT2i) have transformed HF management over the past six years. Initially developed as glucose-lowering agents, they demonstrated robust cardiorenal benefits in the landmark DAPA-HF and EMPEROR-Reduced trials in patients with HF with reduced ejection fraction (HFrEF), irrespective of diabetes status [4], [5]. Subsequent large randomised trials extended these benefits to HF with preserved ejection fraction (HFpEF) and mildly reduced ejection fraction (HFmrEF), principally through reductions in worsening HF events and HF hospitalisations [6], [7]. Reflecting this expanding evidence base, the 2023 focused update of the European Society of Cardiology (ESC) HF guidelines upgraded dapagliflozin and empagliflozin to a Class I, Level A recommendation in HFmrEF and HFpEF, complementing their established position in HFrEF [2], [8]. SGLT2i thereby became the first pharmacological class recommended across the entire ejection fraction spectrum in HF.

Subgroup analyses from major trials have demonstrated preserved efficacy of SGLT2i across age strata, including patients above 75 years, and dedicated analyses in patients with higher frailty burden have similarly suggested maintained benefit [9], [10], [11], [12]. However, trial populations remain younger and less clinically complex than real-world cardiogeriatric cohorts, limiting direct extrapolation to very old patients, particularly octogenarians and nonagenarians with severe cognitive impairment, advanced CKD, frailty, multimorbidity, or palliative-oriented care plans [13], [14]. Available real-world prescribing studies have reported incomplete uptake of SGLT2i following incorporation into HF guidelines, particularly among older or clinically complex patients, with prescription rates remaining substantially below guideline expectations in several contemporary cohorts [15], [16], [17], [18]. Data are therefore scarce in populations predominantly composed of adults aged 80 years and older and are particularly limited in specialised cardiogeriatric settings. The present study was designed to characterise real-world prescription patterns of SGLT2i at discharge after HF hospitalisation in a very old cardiogeriatric population. The primary objective was to identify the clinical, renal, geriatric, and therapeutic factors associated with SGLT2i non-prescription at discharge in a very old population with HF. Particular attention was given to renal dysfunction, markers of disease severity and biological vulnerability, palliative-oriented care, overall pharmacological treatment intensity, and HF phenotype. Secondary objectives were to examine prescription patterns according to diabetes status and glycaemic control. We hypothesised that non-prescription would be more strongly associated with renal dysfunction, clinical complexity, and goals-of-care considerations than with diabetes status or HbA1c level.

## 2 Methods

### 2.1 Study design and setting

This was a single-centre retrospective cross-sectional study based on routinely collected data from a specialised acute cardiogeriatric care unit at Hôpital La Porte Verte, Versailles, France. The unit specialises in the management of older adults with cardiovascular disease. Medication reconciliation and discharge prescription review were routinely performed by the treating medical team, with pharmacist involvement when clinically indicated. No study-specific prescribing protocol or mandatory SGLT2i eligibility checklist was used. Eligible hospitalisations occurred between January 1, 2025, and February 13, 2026. The start date corresponded to the implementation of standardised collection of the variables required for the present analysis, and February 13, 2026, was the date of the final database extraction. The study period was therefore determined by the availability and completeness of routinely collected data and was defined independently of the study results. The study was reported in accordance with the STROBE and RECORD statements [19], [20].

Each hospitalisation included in the analysis corresponded to a unique patient; therefore, the hospitalisation-level and patient-level analyses were identical in the present dataset.

### 2.2 Eligibility and study population

**Fig. 1:**
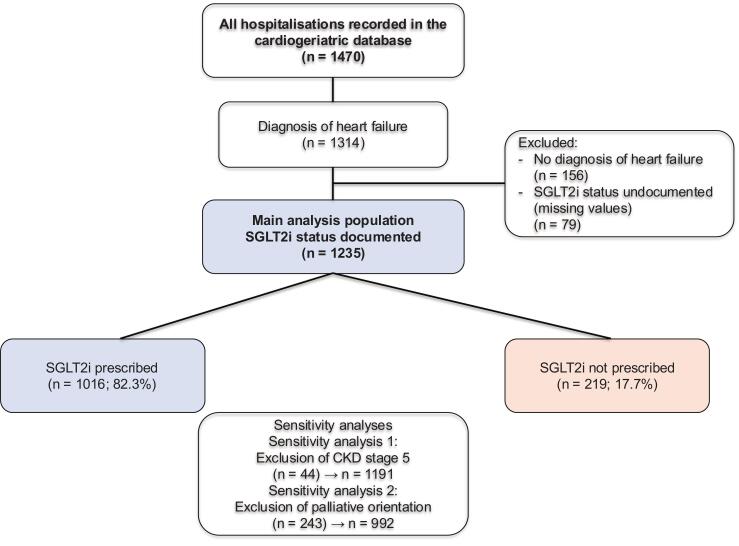
Study flow chart. Of 1470 hospitalisations recorded in the institutional database, 1235 met inclusion criteria and constituted the main analysis study population (SGLT2i prescribed: *n* = 1016 [82.3%]; not prescribed: *n* = 219 [17.7%]). Two sensitivity analyses were prespecified: exclusion of hospitalisations with CKD stage 5 (*n* = 44) and exclusion of hospitalisations with palliative orientation (*n* = 243). Abbreviations: CKD, chronic kidney disease; SGLT2i, sodium–glucose cotransporter 2 inhibitor. Alt text: Study flow chart showing the selection of the main analysis study population. Among 1470 hospitalisations recorded in the institutional cardiogeriatric database, 1314 had a diagnosis of heart failure. After exclusion of 156 hospitalisations without heart failure and 79 with undocumented SGLT2i discharge status, 1235 hospitalisations were included in the main analysis study population: 1016 with SGLT2i prescribed and 219 without SGLT2i prescribed. Two sensitivity analyses excluded CKD stage 5 hospitalisations and palliative-oriented hospitalisations, resulting in analysis populations of 1191 and 992 hospitalisations, respectively.

All hospitalisations with a confirmed HF diagnosis were eligible. The only additional inclusion criterion was documented SGLT2i prescription status at discharge. No formal lower age threshold was applied, as the unit admits patients of all ages with cardiovascular disease requiring geriatric co-management. Hospitalisations with no HF diagnosis (*n* = 156) or undocumented SGLT2i status at discharge (*n* = 79) were excluded. The main analysis study population comprised 1235 hospitalisations (Fig. 1).

### 2.3 Primary outcome

The primary outcome was the absence of an SGLT2i on the discharge prescription. This outcome was intended to characterise SGLT2i prescription at discharge in routine care and should not be interpreted as a measure of inappropriate non-prescription, since formal contraindications, patient preference, and clinician-documented reasons for withholding treatment were not systematically available. As data on pre-admission SGLT2i treatment were unavailable, this outcome captures the presence or absence of an SGLT2i on the discharge order without distinguishing non-initiation from non-resumption or discontinuation.

### 2.4 Variables

Demographic variables included age (continuous), sex, body mass index, living situation, and social isolation. Comorbidity burden was quantified using the Charlson Comorbidity Index (CCI) [21]. Geriatric profile variables included the Activities of Daily Living (ADL) score, [22] severe cognitive impairment, depressive syndrome, malnutrition, and palliative orientation. Palliative orientation was defined as a documented treatment plan prioritising comfort-focused or symptom-oriented care, based on the treating physician's assessment and/or documented goals-of-care discussions, irrespective of formal involvement of a specialist palliative care team. Cardiac characterisation included LVEF category (HFrEF ≤40%, HFmrEF 41–49%, or HFpEF ≥50%, defined according to the 2021 ESC guidelines [1]) and valvular findings. Renal function was characterised as CKD stage at discharge, modelled as a three-level categorical variable (stages 1–3B, stage 4, and stage 5), available for 1234/1235 hospitalisations (99.9%). Exact estimated glomerular filtration rate values were not available in the database; the analysis therefore evaluates real-world prescribing patterns rather than strictly regulatory eligibility. CKD stages were defined according to the KDIGO classification as recorded in the institutional database: stage 1 (eGFR >90 mL/min/1.73m^2^), stage 2 (60–89), stage 3 A (45–59), stage 3B (30–44), stage 4 (15–29), and stage 5 (<15 mL/min/1.73m^2^). For multivariable modelling, stages 1 through 3B were collapsed into a single reference category, stage 4 was treated as the first elevated stratum, and stage 5 as the highest stratum of renal impairment. Laboratory variables included albumin, ferritin, transferrin saturation, moderate-to-severe anaemia, defined as haemoglobin <10 g/dL, NT-proBNP at admission and discharge, and HbA1c (restricted to diabetic patients). Discharge treatment variables included beta-blocker, ACEi/ARB, ARNi, MRA, SGLT2i, diuretic, anticoagulant, and total number of medications.

### 2.5 Statistical analysis

Continuous variables are reported as median [interquartile range, IQR]; categorical variables as counts and percentages. Between-group comparisons used the Mann–Whitney *U* test for continuous variables and the chi-squared or Fisher exact test, as appropriate, for categorical variables. All tests were two-sided, with *p* < 0.05 considered statistically significant.

A multivariable logistic regression model was fitted with the absence of SGLT2i prescription as the dependent variable. Variables were selected a priori based on clinical relevance and prior evidence, without stepwise procedures. Model A (primary model) included: age, sex, LVEF category, CKD stage (3-level categorical, reference: stages 1–3B), diabetes status, severe cognitive impairment, albumin, Charlson CCI, total number of medications, and palliative orientation. Model B (secondary model) additionally included beta-blocker prescription at discharge, included as a marker of overall guideline-directed therapeutic intensity rather than as a direct clinical barrier to SGLT2i use. Continuous variables were standardised (per 1 standard deviation [SD]). Because diabetes mellitus and chronic kidney disease contribute to the Charlson Comorbidity Index, multicollinearity was assessed using variance inflation factors (VIF) for Models A and B. A supplementary analysis excluding the Charlson Comorbidity Index from Model A was also performed to assess the stability of the estimated associations (Supplementary Table S1). The primary analysis was performed on complete cases, with 14/1235 hospitalisations (1.1%) excluded due to missing albumin, the only variable with missing data.

Because discharge NT-proBNP differed substantially between patients with and without an SGLT2i prescription, a supplementary model additionally included natural log-transformed discharge NT-proBNP. This variable was not included in the prespecified primary model because it reflects several overlapping determinants, including HF severity, congestion, renal dysfunction, and haemodynamic status. Its inclusion also marginally reduced the number of complete cases (*n* = 1218 vs *n* = 1221) (Supplementary Table S2).

Model discrimination was assessed by the area under the receiver operating characteristic curve (AUC). The primary analysis consisted of the multivariable logistic regression model identifying factors independently associated with SGLT2i non-prescription at discharge. Secondary analyses included: (1) SGLT2i prescription rates stratified by LVEF category and diabetes status; (2) a post hoc exploratory description of SGLT2i prescription rates across HbA1c tertiles among patients with diabetes; and (3) an exploratory HFrEF-specific analysis of the overlap between SGLT2i prescription and the prescription of beta-blockers, ACEi/ARB/ARNi, and MRA. The HbA1c analysis was considered descriptive and hypothesis-generating. In the HFrEF subgroup, the number and combinations of guideline-recommended drug classes prescribed at discharge were described according to SGLT2i prescription status. For this analysis, the RAAS-treatment pillar was defined as prescription of either an ACEi/ARB or an ARNi. ACEi/ARB without ARNi and ARNi were treated as mutually exclusive subcategories, and their sum constituted the overall ACEi/ARB/ARNi category. Because systolic blood pressure, serum potassium, symptomatic hypotension, prior drug intolerance, treatment doses, and clinician-documented reasons for withholding treatment were unavailable, this analysis was considered descriptive and hypothesis-generating. Sensitivity analyses were performed by excluding CKD stage 5 hospitalisations (*n* = 44) and by excluding hospitalisations with palliative orientation (*n* = 243). All analyses were performed using R version 4.5.1 with the pROC and car packages.

### 2.6 Ethics

This retrospective cross-sectional study was conducted using pseudonymised routinely collected healthcare data, with no additional intervention, examination, or patient contact for research purposes. According to institutional assessment and the French regulatory framework applicable to retrospective studies based on reuse of routine care data, the study was classified as research not involving human participants (recherche n'impliquant pas la personne humaine, RNIPH) and therefore did not require review by a Comité de Protection des Personnes or individual written informed consent. Data processing was conducted in accordance with institutional procedures applicable to the MR-004 reference methodology of the French Data Protection Authority (CNIL).

## 3 Results

### 3.1 Study population and patient flow

The study included 1470 hospitalisations. After exclusion of 156 without a confirmed HF diagnosis and 79 with undocumented SGLT2i discharge status, the main analysis study population comprised 1235 hospitalisations (Fig. 1). Among these, 1016 (82.3%) had an SGLT2i prescribed at discharge, and 219 (17.7%) did not.

### 3.2 Baseline characteristics

The study population was characterised by very advanced age, with a median age of 88.6 years [IQR 82–92] (Table 1). A total of 152 patients (12.3%) were aged 75 to 79 years, 1019 (82.5%) were aged 80 years or older, and 64 (5.2%) were aged under 75 years, reflecting the unselected nature of admissions to the cardiogeriatric unit. SGLT2i prescription rates were 82.1% among patients aged 75 to 79 years and 82.6% among those aged 80 years or older. Women accounted for 54.5% of hospitalisations, and diabetes mellitus was present in 32.7%. Comorbidity burden was high, with a median Charlson CCI of 8 [IQR 7–9]. Geriatric vulnerability was frequent: severe cognitive impairment in 18.4%, malnutrition in 23.0%, and palliative orientation in 19.7% of hospitalisations. HFpEF was the most common phenotype (58.2%), followed by HFrEF (29.2%) and HFmrEF (11.6%).

**Table 1:** Baseline characteristics, clinical profile, and discharge treatment according to SGLT2 inhibitor prescription status.

Variable	All	No SGLT2i	SGLT2i	*p*-value
	(*n* = 1235)	(*n* = 219)	(n = 1016)	
**Demographics**				
Age, median [IQR], years	88.6 [82–92]	89.3 [82–93]	88.5 [83–92]	0.252
Female sex, n (%)	673 (54.5%)	104 (47.5%)	569 (56.0%)	0.026
BMI, median [IQR], kg/m^2^	23.6 [20.5–27.5]	23.9 [20.1–27.1]	23.5 [20.5–27.5]	0.828
Nursing home	144 (11.7%)	33 (15.1%)	111 (10.9%)	0.106
Social isolation	64 (5.2%)	16 (7.3%)	48 (4.7%)	0.163

**Geriatric Profile**				
Charlson CCI, median [IQR]	8 [7–9]	8 [7–10]	8 [7–9]	0.001
ADL score, median [IQR]	5.5 [4.5–6.0]	5.5 [4.5–6.0]	5.5 [4.5–6.0]	0.225
Severe cognitive impairment	227 (18.4%)	58 (26.5%)	169 (16.6%)	0.001
Malnutrition	284 (23.0%)	43 (19.6%)	241 (23.7%)	0.224
Depressive syndrome	283 (22.9%)	55 (25.1%)	228 (22.4%)	0.444
Palliative orientation	243 (19.7%)	64 (29.2%)	179 (17.6%)	< 0.001
Obesity	147 (11.9%)	30 (13.7%)	117 (11.5%)	0.430

**Cardiovascular Comorbidities**				
Hypertension	827 (67.0%)	142 (64.8%)	685 (67.4%)	0.511
Diabetes mellitus	404 (32.7%)	79 (36.1%)	325 (32.0%)	0.276
Atrial fibrillation/flutter	897 (72.6%)	158 (72.1%)	739 (72.7%)	0.925
Ischaemic heart disease	75 (6.1%)	13 (5.9%)	62 (6.1%)	1.000
History of stroke	201 (16.3%)	28 (12.8%)	173 (17.0%)	0.149
Peripheral artery disease	138 (11.2%)	27 (12.3%)	111 (10.9%)	0.631
COPD	129 (10.4%)	26 (11.9%)	103 (10.1%)	0.523
Cardiac amyloidosis	24 (1.9%)	9 (4.1%)	15 (1.5%)	0.022
Dyslipidaemia	790 (64.0%)	148 (67.6%)	642 (63.2%)	0.250
Active cancer	83 (6.7%)	18 (8.2%)	65 (6.4%)	0.408

**Left Ventricular Ejection Fraction (p < 0.001 overall)**				
HFrEF (EF ≤40%)	361 (29.2%)	76 (34.7%)	285 (28.1%)	
HFmrEF (EF 41–49%)	143 (11.6%)	21 (9.6%)	122 (12.0%)	
HFpEF (EF ≥50%)	719 (58.2%)	114 (52.1%)	605 (59.5%)	

**Valves and Devices**				
At least moderate aortic stenosis	194 (15.7%)	37 (16.9%)	157 (15.5%)	0.667
At least moderate mitral regurgitation	264 (21.4%)	42 (19.2%)	222 (21.9%)	0.433
At least moderate tricuspid regurgitation	238 (19.3%)	34 (15.5%)	204 (20.1%)	0.146
Pacemaker / ICD	280 (22.7%)	48 (21.9%)	232 (22.8%)	0.838

**Renal Function at Discharge — CKD Stage (p < 0.001 overall)**				
Stage 1	11 (0.9%)	1 (0.5%)	10 (1.0%)	
Stage 2	236 (19.1%)	32 (14.6%)	204 (20.1%)	
Stage 3A	263 (21.3%)	33 (15.1%)	230 (22.6%)	
Stage 3B	375 (30.4%)	40 (18.3%)	335 (33.0%)	
Stage 4	304 (24.6%)	81 (37.0%)	223 (21.9%)	
Stage 5	44 (3.6%)	32 (14.6%)	12 (1.2%)	

**Laboratory**				
Albumin, median [IQR], g/L †	32.8 [29.4–36.0]	32.0 [28.5–35.7]	32.9 [29.7–36.0]	0.062
Moderate-to-severe anaemia (Hb <10 g/dL)	265 (21.5%)	62 (28.3%)	203 (20.0%)	0.008
True iron deficiency	325 (26.3%)	57 (26.0%)	268 (26.4%)	0.982
NT-proBNP at admission, pg/mL	3818 [1533–8408]	5728 [2126–14,129]	3508 [1471–7535]	< 0.001
NT-proBNP at discharge, pg/mL	2501 [1064–5811]	4619 [1821–11,662]	2279 [951–5099]	< 0.001

**Treatment at Discharge**				
Beta-blocker	794 (64.3%)	101 (46.1%)	693 (68.2%)	< 0.001
ACEi / ARB	325 (26.3%)	43 (19.6%)	282 (27.8%)	0.017
ARNi	157 (12.7%)	8 (3.7%)	149 (14.7%)	< 0.001
MRA	246 (19.9%)	42 (19.2%)	204 (20.1%)	0.834
Diuretic	1130 (91.5%)	186 (84.9%)	944 (92.9%)	< 0.001
Diuretic dose, mg furosemide equivalent	80 [80–125]	125 [40–250]	80 [80–125]	0.026
Anticoagulant	887 (71.8%)	143 (65.3%)	744 (73.2%)	0.022
Number of medications, median [IQR]	8 [6–9]	7 [5–9]	8 [6–10]	< 0.001
Length of stay, days, median [IQR]	7 [6–10]	8 [6–12]	7 [6–9]	0.018

Abbreviations: ACEi, angiotensin-converting enzyme inhibitor; ADL, Activities of Daily Living; ARB, angiotensin receptor blocker; ARNi, angiotensin receptor–neprilysin inhibitor; BMI, body mass index; CCI, Charlson Comorbidity Index; CKD, chronic kidney disease; COPD, chronic obstructive pulmonary disease; EF, ejection fraction; Hb, haemoglobin; HFmrEF, heart failure with mildly reduced ejection fraction; HFpEF, heart failure with preserved ejection fraction; HFrEF, heart failure with reduced ejection fraction; ICD, implantable cardioverter-defibrillator; IQR, interquartile range; MRA, mineralocorticoid receptor antagonist; NT-proBNP, N-terminal pro-B-type natriuretic peptide; SGLT2i, sodium–glucose cotransporter 2 inhibitor.

Continuous variables are expressed as median [interquartile range]; categorical variables as n (%). Between-group comparisons used the Mann–Whitney U test for continuous variables and the chi-squared or Fisher exact test for categorical variables. † Albumin available in 1221/1235 hospitalisations (98.9%).

Compared with hospitalisations receiving SGLT2i, those without SGLT2i at discharge had significantly higher rates of severe cognitive impairment (26.5% vs 16.6%, *p* = 0.001), palliative orientation (29.2% vs 17.6%, *p* < 0.001), and moderate-to-severe anaemia (28.3% vs 20.0%, *p* = 0.008). CKD stage distribution differed markedly (*p* < 0.001): CKD stage 4 was present in 37.0% of non-prescribed vs 21.9% of prescribed hospitalisations, and CKD stage 5 in 14.6% vs 1.2%, respectively. NT-proBNP at admission was substantially higher in the non-prescribed group (5728 [IQR 2126–14,129] vs 3508 [1471–7535] pg/mL, *p* < 0.001), as was NT-proBNP at discharge (4619 [1821–11,662] vs 2279 [951–5099] pg/mL, p < 0.001). The median number of medications was lower in the non-prescribed group (7 [IQR 5–9] vs 8 [6], [7], [8], [9], [10], p < 0.001), and length of stay was slightly longer (8 [6], [7], [8], [9], [10], [11], [12] vs 7 [6], [7], [8], [9] days, *p* = 0.018). Diabetes mellitus did not differ significantly between groups (36.1% vs 32.0%, *p* = 0.276).

### 3.3 SGLT2i prescription across HF phenotypes and diabetes status

**Fig. 2:**
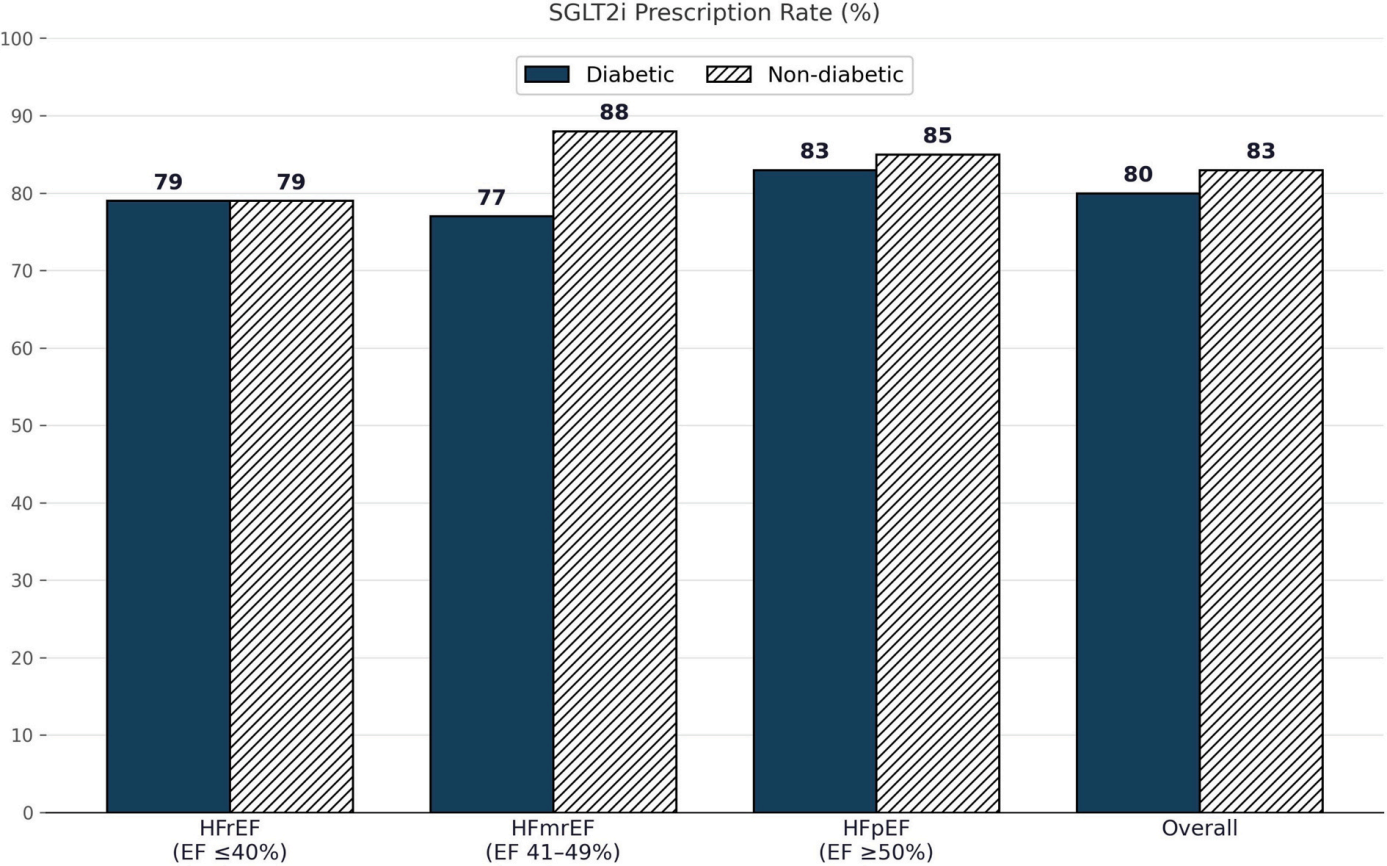
SGLT2 inhibitor prescription across HF phenotype and diabetes status. Overall prescription rate was 82.3%. Rates by LVEF category: HFrEF 78.9%, HFmrEF 85.3%, HFpEF 84.1% (*p* < 0.001 overall). Prescription did not differ significantly between diabetic and non-diabetic patients (80.4% vs 83.2%, *p* = 0.276). In the primary multivariable model, diabetes was not independently associated with non-prescription (aOR 1.35 [95% CI 0.92–1.99], *p* = 0.122). Abbreviations: aOR, adjusted odds ratio; CI, confidence interval; EF, ejection fraction; HFmrEF, heart failure with mildly reduced ejection fraction; HFpEF, heart failure with preserved ejection fraction; HFrEF, heart failure with reduced ejection fraction; NS, not significant; SGLT2i, sodium–glucose cotransporter 2 inhibitor. Alt text: Grouped bar chart showing SGLT2i prescription rates according to left ventricular ejection fraction category and diabetes status. Prescription rates were 79% in diabetic and 79% in non-diabetic patients with HFrEF, 77% and 88% in HFmrEF, 83% and 85% in HFpEF, and 80% and 83% overall, respectively.

The overall SGLT2i prescription rate was 82.3% (Fig. 2). Rates differed across LVEF categories: 78.9% in HFrEF, 85.3% in HFmrEF, and 84.1% in HFpEF (*p* < 0.001 overall). Prescription did not differ significantly between diabetic and non-diabetic patients (80.4% vs 83.2%, p = 0.276), despite the dual cardio-metabolic indication in diabetic patients. In a post hoc exploratory analysis restricted to the 388 patients with diabetes and available HbA1c data, SGLT2i prescription rates were similar across HbA1c tertiles (80.3%, 83.6%, and 78.9%, respectively; *p* = 0.618).

### 3.4 SGLT2i prescription according to renal function

**Fig. 3:**
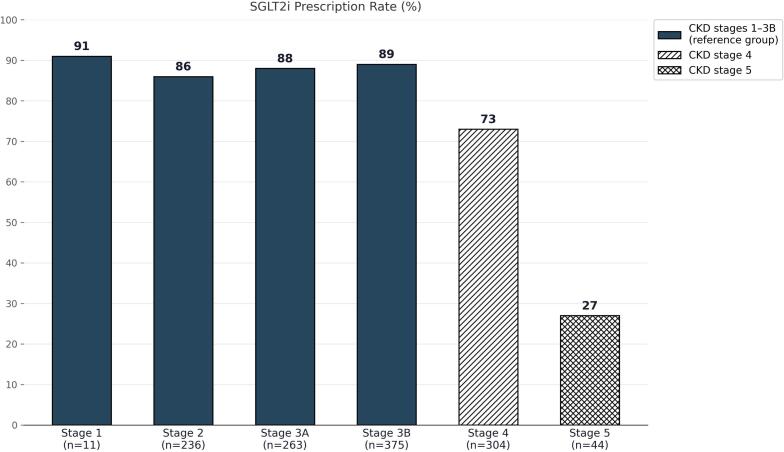
SGLT2 inhibitor prescription according to renal function. Prescription rates were high and stable across CKD stages 1 to 3B (86–91%), declined substantially at stage 4 (73%), and fell sharply at stage 5 (27%; p < 0.001 overall). CKD stages were defined according to the KDIGO classification: stage 1 (eGFR >90 mL/min/1.73m^2^), stage 2 (60–89), stage 3 A (45–59), stage 3B (30–44), stage 4 (15–29), stage 5 (<15 mL/min/1.73m^2^). Abbreviations: CKD, chronic kidney disease; eGFR, estimated glomerular filtration rate; KDIGO, Kidney Disease: Improving Global Outcomes; SGLT2i, sodium–glucose cotransporter 2 inhibitor. Alt text: Bar chart showing SGLT2i prescription rates according to chronic kidney disease stage at discharge. Prescription rates remained high across CKD stages 1 to 3B, ranging from 86% to 91%, then decreased to 73% in stage 4 and 27% in stage 5. Individual bars are distinguished by their fill patterns and labels, with diagonal hatching for CKD stage 4 and crosshatching for CKD stage 5.

A pronounced inverse gradient was observed between CKD stage and SGLT2i prescription (Fig. 3). Prescription rates ranged from 86.4% to 90.9% across CKD stages 1 to 3B, fell to 73.4% in CKD stage 4, and declined sharply to 27.3% in CKD stage 5 (*p* < 0.001 overall).

### 3.5 SGLT2i prescription and other guideline-recommended drug classes in HFrEF

**Fig. 4:**
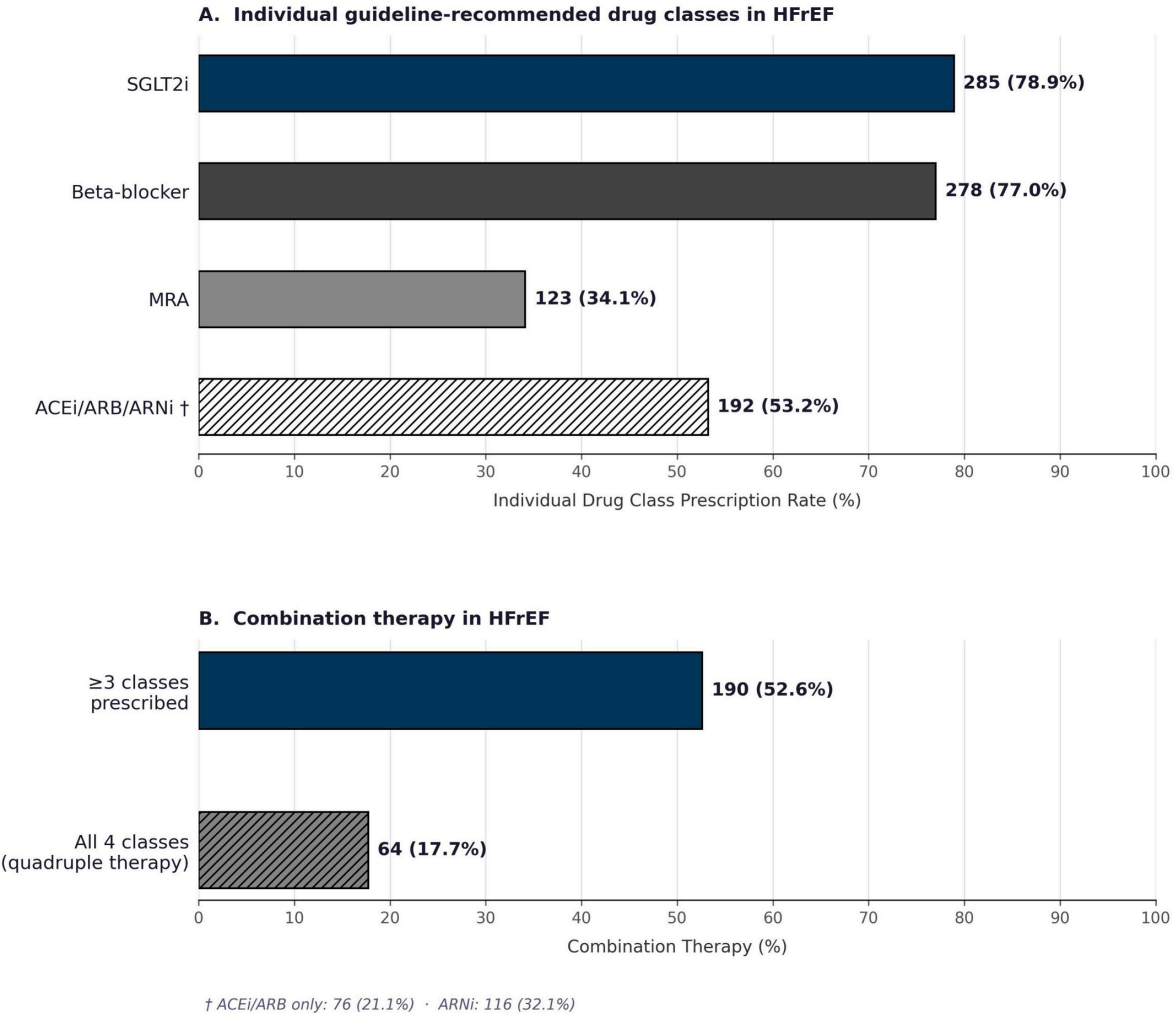
Guideline-recommended drug classes and combination therapy at discharge among patients with HFrEF (*n* = 361). Panel A: individual drug class prescription rates. Panel B: combination therapy rates. Absolute numbers and percentages are shown for each category. Abbreviations: ACEi, angiotensin-converting enzyme inhibitor; ARB, angiotensin receptor blocker; ARNi, angiotensin receptor-neprilysin inhibitor; HFrEF, heart failure with reduced ejection fraction; LVEF, left ventricular ejection fraction; MRA, mineralocorticoid receptor antagonist; SGLT2i, sodium-glucose cotransporter 2 inhibitor. Alt text: Two-panel horizontal bar chart showing guideline-recommended heart failure therapy at discharge among patients with HFrEF. The upper panel shows individual drug-class prescription rates: SGLT2i 79%, beta-blocker 77%, ACEi/ARB/ARNi 53%, and MRA 34%. The lower panel shows combination-therapy rates: 53% received at least three classes and 18% received all four classes.

Among the 361 patients with HFrEF, SGLT2i and beta-blockers were the most frequently prescribed guideline-recommended drug classes at discharge, in 285 (78.9%) and 278 (77.0%) patients, respectively. ACEi/ARB/ARNi were prescribed in 192 patients (53.2%), comprising 76 patients (21.1%) receiving ACEi/ARB without ARNi and 116 patients (32.1%) receiving ARNi. Overall, 126 patients (34.9%) received three of the four guideline-recommended drug classes and 64 (17.7%) received all four classes; thus, 190 patients (52.6%) received at least three classes (Fig. 4).

Compared with patients not receiving an SGLT2i, those receiving an SGLT2i were more frequently prescribed beta-blockers (83.9% vs 51.3%, *p* < 0.001) and ACEi/ARB/ARNi (62.5% vs 18.4%, p < 0.001), whereas MRA prescription rates were similar (34.7% vs 31.6%, *p* = 0.704). Among patients receiving an SGLT2i, 23 (8.1%) received no other guideline-recommended class, whereas 13 patients (17.1%) not receiving an SGLT2i received none of the four drug classes. Detailed treatment patterns according to SGLT2i prescription status are presented in Table 3.

### 3.6 Multivariable analysis of determinants of SGLT2i non-prescription

The primary multivariable analysis was conducted on 1221 complete cases (14 excluded for missing albumin, 1.1%), comprising 214 events (non-prescription) and 1007 non-events. Five of the 14 excluded hospitalisations belonged to the non-prescribed group.

Model A achieved an AUC of 0.750 and Model B an AUC of 0.762 (Table 2).

**Table 2:** Multivariable and sensitivity analyses of factors associated with SGLT2 inhibitor non-prescription at discharge.

Variable	Model A (primary)			Model B (+ beta-blocker)			SA1: excl. CKD stage 5			SA2: excl. Palliative care		
	aOR	95% CI	p	aOR	95% CI	p	aOR	95% CI	p	aOR	95% CI	p
**Renal Function (ref = CKD stages 1–3B)**												
CKD stage 4 (eGFR 15–29 mL/min/1.73m^2^)	**2.79**	1.97–3.96	**<0.001**	**2.77**	1.95–3.95	**<0.001**	**2.82**	1.99–4.00	**<0.001**	**2.95**	1.96–4.42	**<0.001**
CKD stage 5 (eGFR <15 mL/min/1.73m^2^)	**20.29**	9.67–42.56	**<0.001**	**18.83**	8.97–39.54	**<0.001**	*–*	*excl.*	*–*	**20.71**	9.15–46.86	**<0.001**

**Geriatric Vulnerability**												
Palliative orientation	**1.62**	1.09–2.41	**0.016**	**1.51**	1.01–2.25	**0.045**	**1.59**	1.06–2.38	**0.024**	*–*	*excl.*	*–*
Higher albumin (per SD, protective)	**0.83**	0.70–0.97	**0.022**	**0.82**	0.70–0.97	**0.021**	**0.84**	0.71–0.99	**0.037**	0.86	0.71–1.03	0.106
Severe cognitive impairment	1.40	0.94–2.10	0.101	1.47	0.98–2.22	0.064	1.49	0.99–2.24	0.057	1.00	0.57–1.74	0.993

**Heart Failure Phenotype (ref = HFpEF)**												
HFrEF (EF ≤40%)	**1.55**	1.08–2.22	**0.017**	**1.83**	1.26–2.66	**0.001**	**1.64**	1.13–2.37	**0.009**	1.27	0.83–1.95	0.269
HFmrEF (EF 41–49%)	0.86	0.49–1.53	0.608	0.98	0.55–1.76	0.955	0.85	0.46–1.54	0.583	0.99	0.52–1.86	0.963

**Pharmacological Intensity**												
No. of medications (per SD, protective)	**0.60**	0.49–0.72	**<0.001**	**0.65**	0.54–0.79	**<0.001**	**0.58**	0.48–0.71	**<0.001**	**0.64**	0.51–0.80	**<0.001**
Beta-blocker at discharge (Model B only)	–	–	–	**0.46**	0.33–0.66	**<0.001**	–	–	–	–	–	–

**Not Independently Associated**												
Diabetes mellitus	1.35	0.92–1.99	0.122	1.36	0.92–2.00	0.120	1.34	0.90–2.00	0.148	1.31	0.84–2.03	0.230
Charlson CCI (per SD)	1.12	0.94–1.35	0.208	1.11	0.93–1.33	0.254	1.11	0.93–1.34	0.253	1.14	0.92–1.41	0.225
Age (per SD)	0.93	0.78–1.10	0.375	0.94	0.79–1.12	0.472	0.93	0.78–1.11	0.416	0.97	0.79–1.18	0.748
Sex (female)	0.73	0.52–1.03	0.072	0.73	0.52–1.03	0.078	0.73	0.52–1.03	0.074	0.72	0.50–1.04	0.083
**Model details**	*n* *=* *1221 Events* *=* *214 AUC* *=* *0.750*			*n* *=* *1221 Events* *=* *214 AUC* *=* *0.762*			*n* *=* *1178 Events* *=* *183 AUC* *=* *0.717*			*n* *=* *980 Events* *=* *151 AUC* *=* *0.736*		

Model A was the prespecified primary model. Model B additionally included beta-blocker prescription as a marker of overall pharmacological treatment intensity. SA1 excluded patients with CKD stage 5 (n = 44); SA2 excluded patients with palliative-oriented care (n = 243). Continuous variables were standardised per 1 SD. Bold values indicate p < 0.05. — indicates the variable was not applicable (excl. = excluded by design for that sensitivity analysis).

Abbreviations: aOR, adjusted odds ratio; AUC, area under the receiver operating characteristic curve; CI, confidence interval; CKD, chronic kidney disease; CCI, Charlson Comorbidity Index; eGFR, estimated glomerular filtration rate; HFmrEF, heart failure with mildly reduced ejection fraction; HFpEF, heart failure with preserved ejection fraction; HFrEF, heart failure with reduced ejection fraction; SA, sensitivity analysis; SD, standard deviation.

**Table 3:** Guideline-recommended therapy at discharge according to SGLT2 inhibitor prescription status among patients with HFrEF.

Variable	Total HFrEF (n = 361)	SGLT2i prescribed (*n* = 285)	SGLT2i not prescribed (*n* = 76)	p-value
**Individual pillar prescription rates**				
SGLT2i	285 (78.9%)	285 (100%)	0 (0%)	–
Beta-blocker	278 (77.0%)	239 (83.9%)	39 (51.3%)	**<0.001**
ACEi/ARB/ARNi (RAAS pillar) †	192 (53.2%)	178 (62.5%)	14 (18.4%)	**<0.001**
— of which ACEi/ARB only	76 (21.1%)	64 (22.5%)	12 (15.8%)	0.242
— of which ARNi	116 (32.1%)	114 (40.0%)	2 (2.6%)	**<0.001**
MRA	123 (34.1%)	99 (34.7%)	24 (31.6%)	0.704

**Number of guideline-recommended classes**				
No pillar prescribed	13 (3.6%)	0 (0%)	13 (17.1%)	**<0.001**
1 class	72 (19.9%)	23 (8.1%)	49 (64.5%)	**<0.001**
2 classes	86 (23.8%)	72 (25.3%)	14 (18.4%)	0.275
3 classes	126 (34.9%)	126 (44.2%)	0 (0%)	**<0.001**
**All 4 classes (quadruple therapy)**	**64 (17.7%)**	**64 (22.5%)**	0 (0%)	–
**≥3 classes prescribed**	**190 (52.6%)**	**190 (66.7%)**	0 (0%)	**<0.001**
SGLT2i as sole guideline class ‡	23 (6.4%)	23 (8.1%)	–	–
No guideline class prescribed §	13 (3.6%)	–	13 (17.1%)	–

Data presented as n (%). Chi-squared test or Fisher exact test as appropriate. The four ESC 2021 Class I guideline-recommended drug classes in HFrEF are SGLT2i, beta-blocker, ACEi/ARB/ARNi (RAAS pillar), and MRA.

† ACEi/ARB/ARNi: the RAAS-treatment pillar was defined as either ACEi/ARB without ARNi or ARNi therapy. These subcategories were mutually exclusive, and their sum constituted the overall ACEi/ARB/ARNi category.

‡ SGLT2i as sole pillar: 23 patients receiving SGLT2i without any other guideline-recommended class (no beta-blocker, no RAAS agent, no MRA).

§ No guideline class: 13 patients not receiving SGLT2i and without any of the other three guideline-recommended classes.

ACEi, angiotensin-converting enzyme inhibitor; ARB, angiotensin receptor blocker; ARNi, angiotensin receptor–neprilysin inhibitor; EF, ejection fraction; HFrEF, heart failure with reduced ejection fraction; MRA, mineralocorticoid receptor antagonist; RAAS, renin–angiotensin–aldosterone system; SGLT2i, sodium–glucose cotransporter 2 inhibitor.

In Model A, higher odds of SGLT2i non-prescription were observed for CKD stage 4 (aOR 2.79, 95% CI 1.97–3.96; *p* < 0.001), CKD stage 5 (aOR 20.29, 95% CI 9.67–42.56; p < 0.001), palliative orientation (aOR 1.62, 95% CI 1.09–2.41; *p* = 0.016), and HFrEF versus HFpEF (aOR 1.55, 95% CI 1.08–2.22; *p* = 0.017). Higher albumin (aOR 0.83 per SD, 95% CI 0.70–0.97; *p* = 0.022) and a higher discharge medication count (aOR 0.60 per SD, 95% CI 0.49–0.72; *p* < 0.001) were associated with lower odds of non-prescription.

Age, diabetes, Charlson Comorbidity Index, HFmrEF, and sex were not significantly associated with the primary outcome. Severe cognitive impairment was also not significantly associated with non-prescription (aOR 1.40, 95% CI 0.94–2.10; *p* = 0.101). In Model B, beta-blocker prescription was associated with lower odds of SGLT2i non-prescription (aOR 0.46, 95% CI 0.33–0.66; p < 0.001), while the remaining estimates were directionally consistent with Model A. Variance inflation factors ranged from 1.02 to 1.33 in Model A and from 1.02 to 1.35 in Model B, providing no evidence of problematic multicollinearity. Exclusion of the Charlson Comorbidity Index from Model A did not materially alter the direction or magnitude of the principal associations (CKD stage 4: aOR 2.87 [2.03–4.05]; CKD stage 5: aOR 21.87 [10.48–45.62]; palliative orientation: aOR 1.65 [1.11–2.44]; albumin: aOR 0.83 [0.70–0.97]; number of medications: aOR 0.61 [0.50–0.73]). Diabetes mellitus was associated with non-prescription in this supplementary model (aOR 1.47, 95% CI 1.02–2.11; *p* = 0.040), whereas it was not significant in the prespecified primary model. This difference indicates some sensitivity of the diabetes estimate to model specification and should be interpreted cautiously. Full results are presented in Supplementary Table S1.

In a supplementary model including natural log-transformed discharge NT-proBNP (*n* = 1218, events = 214), discharge NT-proBNP was independently associated with SGLT2i non-prescription (aOR 1.44, 95% CI 1.19–1.74; *p* < 0.001). The associations for CKD stage 4 (aOR 2.51 [1.76–3.58]) and CKD stage 5 (aOR 15.49 [7.24–33.14]) were attenuated but remained significant. Palliative orientation remained significant (aOR 1.51 [1.01–2.25]; *p* = 0.045). The association with HFrEF was no longer significant (aOR 1.16, 95% CI 0.78–1.71; *p* = 0.469), and albumin was no longer independently associated with non-prescription (aOR 0.87, 95% CI 0.74–1.03; *p* = 0.108). Full results are presented in Supplementary Table S2.

### 3.7 Sensitivity analyses

After exclusion of the 44 CKD stage 5 hospitalisations (Sensitivity Analysis 1, *n* = 1191, AUC = 0.717), the non-prescription rate was 15.5%. The primary pattern of associations was preserved: CKD stage 4 (aOR 2.82, 95% CI 1.99–4.00, *p* < 0.001), palliative orientation (aOR 1.59, 95% CI 1.06–2.38, *p* = 0.024), HFrEF phenotype (aOR 1.64, 95% CI 1.13–2.37, *p* = 0.009), higher albumin (aOR 0.84, *p* = 0.037), and higher number of medications (aOR 0.58, *p* < 0.001) all retained independent associations. Diabetes remained non-significant (aOR 1.34, *p* = 0.148).

After exclusion of the 243 hospitalisations with palliative orientation (Sensitivity Analysis 2, *n* = 992, AUC = 0.736), the non-prescription rate was 15.4%. Advanced CKD remained the dominant determinant: CKD stage 4 (aOR 2.95, 95% CI 1.96–4.42, p < 0.001) and CKD stage 5 (aOR 20.71, 95% CI 9.15–46.86, p < 0.001). A higher number of medications remained protective (aOR 0.64, p < 0.001). Notably, HFrEF phenotype (*p* = 0.269), albumin (*p* = 0.106), and severe cognitive impairment (*p* = 0.993) were no longer independently significant, suggesting that these signals were at least partly concentrated in hospitalisations with palliative-oriented care. Diabetes remained non-significant (aOR 1.31, *p* = 0.230). Full multivariable and sensitivity analysis results are presented in Table 2.

## 4 Discussion

In this real-world study population of 1235 HF hospitalisations in a specialised acute cardiogeriatric care setting, SGLT2i were prescribed at discharge in more than four out of five hospitalisations despite a median age approaching 89 years and a high burden of multimorbidity, renal dysfunction, and geriatric vulnerability. SGLT2i prescription rates were similar according to diabetes status and glycaemic control in unadjusted analyses, and diabetes was not associated with non-prescription in the prespecified primary model. However, the diabetes estimate was sensitive to exclusion of the Charlson Comorbidity Index. In the primary model, non-prescription was associated with advanced CKD, palliative orientation, lower albumin, HFrEF phenotype, and lower overall pharmacological intensity. In supplementary analyses, the associations with diabetes, albumin, and HFrEF varied according to model specification, whereas advanced CKD and palliative orientation remained comparatively consistent. These findings describe real-world prescribing patterns in a specialised cardiogeriatric setting but do not determine whether individual decisions to withhold treatment were clinically appropriate.

### 4.1 A high SGLT2i prescription rate in a very old population

The observed prescription rate of 82.3% was higher than rates reported in several early post-guideline observational studies [15], [16], [17], [18], but it should be interpreted in the context of more recent data showing substantial increases in SGLT2i prescription over time. Contemporary data from the Swedish Heart Failure Registry reported prescription rates approaching 80% to 90% across HF phenotypes after broader adoption of updated guideline recommendations [23]. The present findings should therefore not be interpreted as uniquely high uptake, but as evidence that similarly high prescription rates can be observed in a substantially older and more clinically vulnerable cardiogeriatric population.

Broader epidemiological analyses have suggested that a large proportion of patients with HF may be theoretically eligible for SGLT2i therapy [24]. However, theoretical eligibility based on trial criteria does not capture all barriers encountered in very old patients in routine care, including acute clinical instability, patient preference, treatment goals, severe multimorbidity, or contraindications that were not systematically documented in the present database.

### 4.2 Prescription according to diabetes status and glycaemic control

SGLT2i prescription rates were similar in patients with and without diabetes in unadjusted analyses (80.4% vs 83.2%, *p* = 0.276), and diabetes was not independently associated with non-prescription in the prespecified primary model. However, diabetes was modestly associated with non-prescription in the supplementary model excluding the Charlson Comorbidity Index. Because diabetes contributes to the Charlson Comorbidity Index, this difference indicates that the adjusted association was sensitive to model specification and to the overlap between these variables [21]. Among patients with diabetes, no gradient in prescription was observed across HbA1c tertiles. Overall, these findings suggest that glycaemic control was not a major determinant of prescription, while the independent association with diabetes itself remains less certain. This prescribing pattern is consistent with the benefits of SGLT2i demonstrated in patients with and without diabetes across the HF spectrum and with current recommendations irrespective of diabetes status [2], [4], [5], [6], [7].

### 4.3 Advanced renal dysfunction was the dominant determinant of non-prescription

CKD stage was by far the strongest independent determinant of SGLT2i non-prescription. CKD stages 4 and 5 were independently associated with higher odds of SGLT2i non-prescription, although the estimate for CKD stage 5 was based on a small subgroup and was imprecise. These findings are both expected and clinically informative. Renal function is directly relevant to SGLT2i eligibility, as product-specific estimated glomerular filtration rate thresholds influence prescribing decisions in routine practice. Randomised evidence has demonstrated cardiorenal benefits of dapagliflozin in patients with chronic kidney disease and an eGFR as low as 25 mL/min/1.73m^2^ [25]. At the time of the study period, initiation below this threshold and in patients with end-stage kidney disease remained limited by the populations studied and by product-specific regulatory guidance. However, exact eGFR values were unavailable in the database, preventing assessment of formal eligibility at the patient level. In addition, data on dialysis status, acute kidney injury during admission, serum potassium, symptomatic hypotension, and diuretic intensity were unavailable, limiting mechanistic interpretation of renal-related non-prescription. The association observed for CKD stage 5 should be interpreted cautiously. This subgroup included only 44 patients, the outcome showed near-complete separation according to prescription status, and the confidence interval was wide. The magnitude of the adjusted odds ratio is therefore imprecise and should not be interpreted as a stable quantitative estimate. Moreover, this association should not be considered a novel clinical determinant because it likely reflects, at least in part, prescribing-label restrictions and the limited eligibility of patients with end-stage kidney disease or dialysis for treatment initiation during the study period. Dialysis status was unavailable, preventing more detailed assessment of the CKD stage 5 subgroup.

The independent association for CKD stage 4 (eGFR approximately 15–29 mL/min/1.73m^2^) is more nuanced and clinically significant. This stratum likely includes patients who may formally qualify for SGLT2i under current label indications, but in whom clinicians may nonetheless withhold therapy because of acute clinical instability or other patient-level considerations not captured in the present database. Whether non-prescription in CKD stage 4 reflected formal ineligibility, acute clinical instability, clinician concern, or another unmeasured factor cannot be determined from the present data. Further studies using exact eGFR values and clinician-documented reasons for treatment decisions are needed to characterise non-prescription in this subgroup.

### 4.4 Palliative-oriented care and albumin

Palliative orientation remained independently associated with SGLT2i non-prescription after multivariable adjustment (aOR 1.62, *p* = 0.016). Care goals may have prioritised comfort, symptom relief, or reduction of treatment burden over long-term event prevention. Withholding SGLT2i may therefore have reflected individualised decision-making, although the reasons for these decisions and their appropriateness could not be assessed. Documented palliative orientation was therefore associated with discharge prescribing patterns in routine cardiogeriatric HF care, a dimension rarely captured in conventional cardiology registries.

Lower albumin was also independently associated with non-prescription (aOR 0.83 per SD, corresponding to approximately 7 g/L, *p* = 0.022). Albumin is not a contraindication to SGLT2i therapy, but in very old hospitalised patients it serves as an integrative biomarker of malnutrition, systemic inflammation, chronic disease burden, and reduced physiological reserve [26], [27]. Its independent association with non-prescription suggests that lower albumin may be interpreted clinically as a marker of broader biological vulnerability and reduced physiological reserve. Albumin was associated with non-prescription in the primary model but was no longer significant after exclusion of patients with palliative-oriented care. This attenuation suggests that the association was at least partly concentrated among patients with palliative-oriented management and should not be interpreted as evidence of an independent graded biological relationship. The association with albumin was also attenuated and no longer statistically significant in the supplementary model including discharge NT-proBNP, further indicating that albumin should not be interpreted as a robust independent determinant across all model specifications.

### 4.5 HFrEF phenotype and SGLT2i non-prescription: a signal likely influenced by clinical complexity

HFrEF phenotype was independently associated with higher odds of SGLT2i non-prescription compared with HFpEF (aOR 1.55, *p* = 0.017), despite the longer-standing and most robustly established evidence base for SGLT2i in HFrEF [4], [5]. This finding should be interpreted cautiously. In unadjusted analyses, SGLT2i prescription was lower in HFrEF (78.9%) than in HFmrEF (85.3%) or HFpEF (84.1%). However, the absolute differences in prescription rates across HF phenotypes were modest, corresponding to 5.2 percentage points between HFrEF and HFpEF and 6.4 percentage points between HFrEF and HFmrEF. The clinical significance of the adjusted association should therefore be interpreted cautiously. Several unmeasured factors may have contributed to the association between HFrEF and SGLT2i non-prescription, including lower blood pressure, advanced renal dysfunction, hyperkalaemia, recurrent decompensation, cachexia, intolerance to other HF therapies, or clinician-perceived haemodynamic instability. None of these explanations can be confirmed from the present dataset. Previous studies have shown that the benefits of SGLT2i are maintained in older patients and across age groups, including in HFrEF and HFpEF populations [10], [28]. The lower prescription rate observed in HFrEF in the present study is therefore more likely to reflect characteristics of this specific clinical population than a weaker evidence base for SGLT2i therapy.

The association between HFrEF and non-prescription was also attenuated and no longer statistically significant after inclusion of discharge NT-proBNP. The HFrEF finding should therefore be interpreted as dependent on model specification and potentially related to differences in HF severity or congestion captured by NT-proBNP.

The HFrEF-specific treatment analysis provides additional context for this finding. Although SGLT2i and beta-blockers were prescribed in approximately four out of five patients with HFrEF, ACEi/ARB/ARNi and MRA were prescribed in 53.2% and 34.1%, respectively. The high beta-blocker prescription rate in this HFrEF subgroup is consistent with their established evidence-based role in patients with reduced ejection fraction [29]. More than half of patients received at least three guideline-recommended drug classes, whereas complete quadruple therapy was prescribed in 17.7%. In very old patients with HFrEF, hypotension, renal dysfunction, hyperkalaemia, cachexia, and treatment intolerance may limit the prescription of multiple guideline-directed therapies [13], [14]. Patients receiving an SGLT2i were also substantially more likely to receive beta-blockers and ACEi/ARB/ARNi, whereas MRA prescription did not differ significantly according to SGLT2i status. This pattern shows that SGLT2i non-prescription was associated with lower overall pharmacological treatment intensity rather than occurring exclusively as an isolated therapeutic decision. However, systolic blood pressure, serum potassium, symptomatic hypotension, prior intolerance, treatment doses, and clinician-documented reasons for withholding individual therapies were unavailable. Differences between drug classes therefore cannot be interpreted as evidence of relative eligibility, tolerability, safety, or quality of care, and this analysis should be regarded as descriptive and hypothesis-generating. The association between HFrEF and SGLT2i non-prescription persisted after exclusion of patients with CKD stage 5 but was no longer significant after exclusion of patients with palliative-oriented care, suggesting that the association was at least partly concentrated among patients with palliative-oriented treatment goals.

### 4.6 SGLT2i non-prescription and overall pharmacological treatment intensity

The inverse association between total number of medications and SGLT2i non-prescription should not be interpreted as an endorsement of polypharmacy. Rather, medication count likely captured a broader tendency toward active pharmacological management versus treatment de-escalation or simplification. The inverse association between beta-blocker prescription and SGLT2i non-prescription in Model B indicates that SGLT2i omission frequently co-occurred with a broader pattern of lower pharmacological treatment intensity [15], [18], [30].

### 4.7 Clinical implications

Several practical implications emerge from this study. First, the high discharge prescription rate shows that physicians working in this specialised cardiogeriatric unit frequently prescribed SGLT2i to very old patients with HF. This prescribing pattern is consistent with available trial subgroup analyses and emerging real-world evidence supporting the clinical use of SGLT2i in older and very elderly patients with HF [31], [32]. However, because treatment safety, tolerability, discontinuation, adherence, and clinical outcomes were not assessed in the present study, this observation should not be interpreted as evidence that SGLT2i therapy was feasible or safe in all potentially eligible patients. Second, the broadly similar prescription rates according to diabetes status and the absence of an HbA1c gradient are consistent with the contemporary use of SGLT2i as HF-directed cardiovascular therapy rather than solely as glucose-lowering treatment.

Residual non-prescription was concentrated among patients with advanced CKD, palliative-oriented care, and lower overall pharmacological treatment intensity. Because the reasons for individual prescribing decisions were unavailable, the study cannot distinguish formal contraindications, temporary clinical instability, patient preference, treatment de-escalation, or potentially modifiable non-prescription. Prospective studies are needed to determine whether structured discharge and post-discharge medication review can identify additional eligible patients without compromising safety or patient-centred treatment goals.

### 4.8 Strengths and relevance to cardiovascular practice

A principal strength of this study is its focus on a population with a median age of 88.6 years and a high prevalence of multimorbidity, cognitive impairment, renal dysfunction, and palliative-oriented care. Although older patients, including adults aged 80 years and older, have been included in major trials and subgroup analyses, populations with this degree of multimorbidity, cognitive impairment, advanced CKD, and palliative-care needs are less frequently characterised in routine-care prescribing studies. The present findings provide a detailed description of discharge prescribing patterns in a specialised cardiogeriatric setting but do not establish treatment efficacy, safety, eligibility, or appropriateness.

### 4.9 Strengths and limitations

Strengths include the focus on a very old population less frequently characterised in routine-care HF prescribing studies, the use of a prospectively collected cardiogeriatric database, contemporaneity with the 2023 ESC guideline update, and prespecified multivariable and sensitivity analyses.

Several limitations should be acknowledged. The retrospective cross-sectional, single-centre design precludes causal interpretation and limits generalisability beyond specialised cardiogeriatric settings. Although no formal lower age threshold was applied, the median age was 88.6 years and the lower quartile was 82 years. The findings therefore primarily reflect prescribing patterns in octogenarians and nonagenarians and may not be directly generalisable to younger adults with HF. Prescription rates observed in this highly specialised unit may therefore not reflect prescribing patterns in general cardiology or non-specialised geriatric settings. Reasons for non-prescription were not directly recorded, preventing differentiation between formal contraindications, acute instability, patient preference, treatment limitation in the context of palliative-oriented care, or clinician inertia. Pre-admission SGLT2i use was unavailable, preventing distinction between non-initiation, non-resumption, and discontinuation. Exact eGFR values were not available, limiting assessment of regulatory eligibility at the patient level. Data regarding dialysis status, symptomatic hypotension, or specific contraindications to SGLT2i initiation were unavailable*,* limiting mechanistic interpretation of renal-related non-prescription in the CKD stage 4 subgroup. Data on systolic blood pressure, serum potassium, prior intolerance, treatment doses, and clinician-documented reasons for withholding ACEi/ARB/ARNi, beta-blockers, or MRA were also unavailable. Consequently, differences in prescription rates across HFrEF drug classes cannot be interpreted as direct evidence of relative eligibility, tolerability, safety, or quality of care. Finally, this study captured discharge prescription only. No follow-up data were available regarding subsequent adherence, discontinuation, renal safety, mortality, rehospitalisation, functional decline, quality of life, or the clinical appropriateness of non-prescription. Therefore, the study cannot determine whether SGLT2i prescription improved outcomes, whether non-prescription was clinically appropriate, or whether residual non-prescription represented avoidable therapeutic inertia. The findings should consequently be interpreted as descriptive data on prescription at discharge rather than evidence of treatment effectiveness, safety, or appropriateness.

## 5 Conclusions

In this specialised cardiogeriatric setting, SGLT2 inhibitors were prescribed at discharge to more than 80% of a predominantly very old HF population, with broadly similar prescription rates according to diabetes status and glycaemic control. Advanced CKD and palliative-oriented care were the most consistent correlates of non-prescription. HFrEF was associated with higher odds of non-prescription in the primary model, although this association was attenuated after adjustment for discharge NT-proBNP. Among patients with HFrEF, 52.6% received at least three guideline-recommended drug classes and 17.7% received complete quadruple therapy. These findings describe contemporary prescribing patterns but cannot determine the eligibility, safety, or appropriateness of individual treatment decisions. Future comparative studies should assess whether structured in-hospital and post-discharge medication-review protocols improve appropriate initiation or reintroduction of SGLT2 inhibitors and other guideline-directed therapies compared with usual care, while preserving safety and patient-centred treatment goals.

The following are the supplementary data related to this article.

Supplementary Table S1 Multicollinearity assessment and supplementary analysis excluding the Charlson Comorbidity Index from Model A.
Continuous variables standardised per 1 SD. Variance inflation factors ranged from 1.02 to 1.33 in Model A and from 1.02 to 1.35 in Model B, indicating no evidence of problematic multicollinearity. Bold values: *p* < 0.05.
† Diabetes mellitus was associated with non-prescription in the supplementary model excluding the Charlson Comorbidity Index (*p* = 0.040), whereas it was not significant in the prespecified primary model. This difference indicates sensitivity of the diabetes estimate to model specification and should be interpreted cautiously.
Abbreviations: aOR: adjusted odds ratio; CI: confidence interval; CKD: chronic kidney disease; CCI: Charlson Comorbidity Index; eGFR: estimated glomerular filtration rate; HFmrEF: heart failure with mildly reduced ejection fraction; HFpEF: heart failure with preserved ejection fraction; HFrEF: heart failure with reduced ejection fraction; SD: standard deviation.

Supplementary Table S2 Supplementary multivariable model including discharge NT-proBNP.
† Discharge NT-proBNP: natural log-transformed. Not included in the prespecified primary model because it reflects several overlapping determinants including HF severity, congestion, renal dysfunction, and haemodynamic status. Three hospitalisations were additionally excluded due to missing discharge NT-proBNP (*n* = 1218 vs *n* = 1221 in Model A).
Continuous variables standardised per 1 SD. Bold values: *p* < 0.05.
Abbreviations: aOR: adjusted odds ratio; CI: confidence interval; CKD: chronic kidney disease; CCI: Charlson Comorbidity Index; eGFR: estimated glomerular filtration rate; HFmrEF: heart failure with mildly reduced ejection fraction; HFpEF: heart failure with preserved ejection fraction; HFrEF: heart failure with reduced ejection fraction; NT-proBNP: N-terminal pro-B-type natriuretic peptide; SD: standard deviation.

## Sponsor's role

No sponsor had any role in the design, analysis, interpretation, or preparation of this manuscript.

## Declaration of generative AI and AI-assisted technologies in the manuscript preparation process

During the preparation of this work, the authors used AI to assist with language editing and manuscript restructuring. After using this tool, the authors reviewed and edited the content as needed and take full responsibility for the content of the published article.

## CRediT authorship contribution statement

**Rémi Esser:** Writing – review & editing, Supervision, Methodology, Investigation, Conceptualization. **Marc Harboun:** Writing – review & editing, Supervision, Methodology. **Marine Larbaneix:** Writing – review & editing, Investigation, Data curation. **Alejandro Mondragon:** Writing – review & editing, Investigation, Data curation. **Sophie Balesdent:** Writing – review & editing, Investigation, Data curation. **Marlène Esteban:** Writing – review & editing, Investigation, Data curation. **Jennifer Corny:** Writing – review & editing, Methodology, Investigation. **Christine Farges:** Writing – review & editing, Project administration, Investigation. **Vincenzo Palermo:** Writing – review & editing, Supervision, Methodology. **Nicolas Pages:** Writing – review & editing, Visualization, Formal analysis, Data curation. **Sophie Nisse Durgeat:** Writing – review & editing, Visualization, Formal analysis, Data curation. **Olivier Maurou:** Writing – review & editing, Writing – original draft, Visualization, Methodology, Formal analysis, Data curation, Conceptualization.

## Ethics

This retrospective cross-sectional study was conducted using pseudonymised routinely collected healthcare data, with no additional intervention, examination, or patient contact for research purposes. All procedures were conducted in accordance with the Declaration of Helsinki, relevant French laws, institutional guidelines, and the MR-004 reference methodology of the French Data Protection Authority (CNIL). According to institutional assessment and the French regulatory framework applicable to retrospective studies based on reuse of routine care data, the study was classified as research not involving human participants (recherche n'impliquant pas la personne humaine, RNIPH) and therefore did not require review by a Comité de Protection des Personnes or individual written informed consent. The privacy rights of patients were observed through pseudonymisation and secure institutional data processing. Data processing was approved according to institutional procedures.

## Funding

This research did not receive any specific grant from funding agencies in the public, commercial, or not-for-profit sectors.

## Declaration of competing interest

Sophie Nisse Durgeat and Nicolas Pages are employed by NP Medical. All other authors declare no competing interests. The authors declare that they have no known competing financial interests or personal relationships that could have appeared to influence the work reported in this paper.

All authors have completed the ICMJE disclosure form and report no conflicts of interest related to this manuscript.

## Data Availability

The data underlying this article cannot be shared publicly due to institutional and regulatory restrictions related to pseudonymised healthcare data. Reasonable requests may be considered by the corresponding author, subject to institutional approval and applicable data protection regulations.
