# Supplementary material for: SGLT2 inhibitor prescription at discharge in very old adults with heart failure irrespective of diabetes status
Source: Int J Cardiol Heart Vasc. 2026 Jul 30;66:101980. doi: 10.1016/j.ijcha.2026.101980 (PMC13448023; doi:10.1016/j.ijcha.2026.101980)
Supplement: Supplementary file 1 [file mmc1.docx]

**Supplementary Table S1.** Multicollinearity assessment and supplementary analysis excluding the Charlson Comorbidity Index from Model A.

| **Variable** | **aOR** | **95% CI** | **p** | **aOR** | **95% CI** | **p** |
| --- | --- | --- | --- | --- | --- | --- |
|  | **Model A (with Charlson)** | | | **Model A — no Charlson** | | |
| **Renal Function** |  |  |  |  |  |  |
| CKD stage 4 (eGFR 15–29 mL/min/1.73m²) | **2.79** | 1.97–3.96 | **<0.001** | **2.87** | 2.03–4.05 | **<0.001** |
| CKD stage 5 (eGFR <15 mL/min/1.73m²) | **20.29** | 9.67–42.56 | **<0.001** | **21.87** | 10.48–45.62 | **<0.001** |
| **Geriatric Vulnerability** |  |  |  |  |  |  |
| Palliative orientation | **1.62** | 1.09–2.41 | **0.016** | **1.65** | 1.11–2.44 | **0.013** |
| Higher albumin (per SD, protective) | **0.83** | 0.70–0.97 | **0.022** | **0.83** | 0.70–0.97 | **0.020** |
| Severe cognitive impairment | 1.40 | 0.94–2.10 | 0.101 | 1.42 | 0.95–2.13 | 0.086 |
| **Heart Failure Phenotype (ref = HFpEF)** |  |  |  |  |  |  |
| HFrEF (EF ≤40%) | **1.55** | 1.08–2.22 | **0.017** | **1.55** | 1.08–2.22 | **0.016** |
| HFmrEF (EF 41–49%) | 0.86 | 0.49–1.53 | 0.608 | 0.87 | 0.49–1.53 | 0.620 |
| **Pharmacological Intensity** |  |  |  |  |  |  |
| No. of medications (per SD, protective) | **0.60** | 0.49–0.72 | **<0.001** | **0.61** | 0.50–0.73 | **<0.001** |
| **Not Independently Associated** |  |  |  |  |  |  |
| Diabetes mellitus | 1.35 | 0.92–1.99 | 0.122 | **1.47** | 1.02–2.11 | **0.040** |
| Charlson CCI (per SD) | 1.12 | 0.94–1.35 | 0.208 | *—* | *excluded* | *—* |
| Age (per SD) | 0.93 | 0.78–1.10 | 0.375 | 0.95 | 0.80–1.12 | 0.550 |
| Sex (female) | 0.73 | 0.52–1.03 | 0.072 | 0.72 | 0.51–1.00 | 0.050 |
| **Model details** | *n=1 221 · Events=214 · AUC=0.750* | | | *n=1 221 · Events=214* | | |
